# Female Sexual Dysfunction Across the Female Lifespan: The Impact of Menopause, Pelvic Floor Disorders, and Pelvic Surgery

**DOI:** 10.3390/jcm15197550

**Published:** 2026-09-28

**Authors:** Andrea Braga, Alison De Souza, Angie Rantell, Luiz Gustavo Brito, Jan-Paul Roovers, Sònia Anglès-Acedo, Maurizio Serati

**Affiliations:** 1 Department of Obstetrics and Gynaecology, Ente Ospedaliero Cantonale-Beata Vergine Hospital, 6850 Mendrisio, Switzerland; 2 Faculty of Biomedical Sciences, Università della Svizzera Italiana, 6900 Lugano, Switzerland; 3 Department of Urogynaecology, Mercy Hospital for Women, 163 Studley Road, Heidelberg, VIC 3084, Australia; adesouza@bigpond.net.au; 4 Department of Urogynaecology, King’s College Hospital London and College of Health, Medicine and Life Sciences, Brunel University, London SE5 9RS, UK; angela.rantell@nhs.net; 5 Department of Obstetrics and Gynaecology, Ribeirão Preto Medical School, University of São Paulo, São Paulo 05508-220, Brazil; lgobrito@gmail.com; 6 Department of Obstetrics and Gynaecology, Amsterdam University Medical Centers, 1100 DD Amsterdam, The Netherlands; j.p.roovers@amsterdamumc.nl; 7 Institut Clínic de Ginecologia, Obstetrícia i Neonatologia, Grup de Treball de Sexologia Clinica, Hospital Clínic de Barcelona, c.Villarroel, 170, 08036 Barcelona, Spain; sangles@ub.edu; 8 Faculty of Medicine, University of Barcelona, 08007 Barcelona, Spain; 9 Institut d’Investigacions Biomèdiques August Pi i Sunyer (IDIBAPS), 08036 Barcelona, Spain; 10 Department of Obstetrics and Gynaecology, Del Ponte Hospital, University of Insubria, 21100 Varese, Italy; mauserati@hotmail.com

**Keywords:** female sexual dysfunction, sexuality, sexual disorders, sexual well-being, quality of life

## Abstract

Female sexual dysfunction is a multifactorial condition influenced by biological, psychological, relational, and sociocultural factors that can compromise sexual well-being. It is a prevalent condition, affecting nearly 40% of the global female population. Furthermore, problems related to desire, arousal and orgasm have an impact on 12% of women across the lifespan. Despite the high prevalence of this issue and its harmful impact on mental health and vitality, a considerable number of women do not seek medical care. Another issue is the lack of physicians specialising in sexual health problems. Indeed, female sexual dysfunction is often misdiagnosed, and even when identification is successful, treatment is not always appropriate. This narrative review aims to address female sexual dysfunction in relation to menopause, pelvic organ prolapse, urinary incontinence, postpartum changes, and pelvic floor surgery. It provides a comprehensive overview of these conditions, focusing on the underlying pathophysiological mechanisms, their impact on female sexual health, and the available preventive strategies for their management.

## 1. Introduction

According to the World Health Organization (WHO), sexual health refers to a state of physical, emotional, mental and social well-being in relation to sexuality. Disorders in any of these areas can affect sexual function with a significant impact on women’s quality of life (QoL) and social interactions.

Female sexual dysfunction (FSD) is a multifactorial condition influenced by biological, psychological, relational, and sociocultural factors. FSD encompasses a group of disorders characterized by clinically significant disturbances in one or more phases of the sexual response cycle, including sexual desire, arousal, and orgasm. According to the International Classification of Diseases, 11th Revision (ICD-11), sexual dysfunctions are defined as persistent difficulties in experiencing personally satisfying, non-coercive sexual activities that occur frequently, are present for several months, and cause significant distress. The main categories include hypoactive sexual desire dysfunction, characterized by reduced or absent sexual interest; sexual arousal dysfunction, involving diminished subjective or physiological sexual arousal despite adequate stimulation; and orgasmic dysfunction, characterized by absent, delayed, infrequent, or less intense orgasm despite adequate sexual stimulation. Other specified and unspecified sexual dysfunctions are recognized when symptoms do not fit these categories [1].

ICD-11 and the Diagnostic and Statistical Manual of Mental Disorders, Fifth Edition (DSM-5) share a biopsychosocial framework for FSD but differ in their diagnostic approach. ICD-11 maintains separate desire and arousal disorders and permits etiological qualifiers, whereas DSM-5 combines these conditions into Female Sexual Interest/Arousal Disorder and excludes dysfunctions entirely attributable to another medical or psychiatric condition [1].

FSD is a prevalent condition, affecting nearly 40% of the global female population [2]. Furthermore, problems related to desire, arousal and orgasm have an impact on 12% of women across the lifespan. A recent systematic review and meta-analysis aimed to examine the prevalence and associated factors of FSD among healthy reproductive-aged women showed that its prevalence ranges between 20% and 95%. Based on 18 studies, a meta-analysis revealed a pooled FSD prevalence of 48% (95% CI: 39.19–56.43%) [3]. In addition, a series of sociodemographic factors (such as higher Body Mass Index (BMI), higher age, higher marriage duration, rural residential environment, etc.) have been identified as risk factors for FSD [3]. The wide variability in prevalence estimates reported in the literature is likely attributable to differences in study methodology, population characteristics, diagnostic criteria, and assessment tools used to investigate FSD. Moreover, FSD remains frequently underreported because of sociocultural, psychological, and relational barriers that may limit disclosure, contributing to the heterogeneity of reported prevalence rates.

Despite the high prevalence of this issue and its harmful impact on mental health and vitality, a considerable number of women do not seek medical care [4]. Another issue is the lack of physicians skilled in sexual health problems. Indeed, FSD is often misdiagnosed, and even when identification is obtained, treatment is not always appropriate. Furthermore, human sexuality may be influenced by a multitude of factors and circumstances in the woman’s life.

This narrative review, supported by a structured literature search, aims to address FSD in relation to menopause, pelvic organ prolapse, urinary incontinence, postpartum changes, and pelvic floor surgery. It provides a comprehensive overview of these conditions, focusing on the underlying pathophysiological mechanisms, their impact on female sexual health, and the available preventive strategies for their management.

## 2. Materials and Methods

This narrative review, supported by a structured literature search, was performed with a cut-off date of 31 May 2026, using the PubMed/MEDLINE and CENTRAL (Cochrane Central Register of Controlled Trials) databases.

### 2.1. Search Strategy

The search strategy included the following combinations of keywords and Medical Subject Headings (MeSH) terms: “female sexual dysfunction”, “sexual well-being”, “sexual dysfunctions (psychological)”, “sexuality”, “sexual disorders”, “menopause”, “pelvic organ prolapse”, “pelvic surgery”, “urinary incontinence”, “postpartum period”, “pelvic floor”, and “quality of life”.

### 2.2. Selection and Appraisal

The following epidemiological study designs were included in the selection process: systematic reviews, meta-analyses, randomised controlled trials, large cohort studies and leading clinical guidelines. Additional relevant articles were identified through manual screening of reference lists. All studies published up to 31 May 2026 were considered for inclusion, with this date serving as the predefined search cut-off. To minimize potential confounding factors and maintain a specific focus on FSD, only studies in which female sexual function (SF) was assessed as a primary outcome were included. Studies evaluating SF solely as a secondary endpoint were excluded to improve the homogeneity of the evidence and ensure a more direct assessment of factors associated with sexual dysfunction. Articles not published in English, case reports, commentaries, editorials, conference abstracts, and studies not relevant to the predefined review themes were excluded.

Although no formal quantitative quality assessment was performed, the included studies were critically appraised according to their methodological design, sample size, population characteristics, scientific relevance, and consistency with the existing literature. Greater emphasis was placed on evidence derived from systematic reviews, meta-analyses, randomized controlled trials, and large observational studies when drawing clinical conclusions. Cross-sectional and retrospective observational studies were also considered when they provided clinically relevant evidence and contributed substantially to the understanding of FSD within the scope of this review. Studies were included if they addressed the prevalence, pathogenesis, and/or treatment of FSD within the specified contexts.

### 2.3. Data Reduction

This phase was conducted using a structured process that reorganised a large and heterogeneous body of evidence into coherent thematic domains.

**Figure 1:**
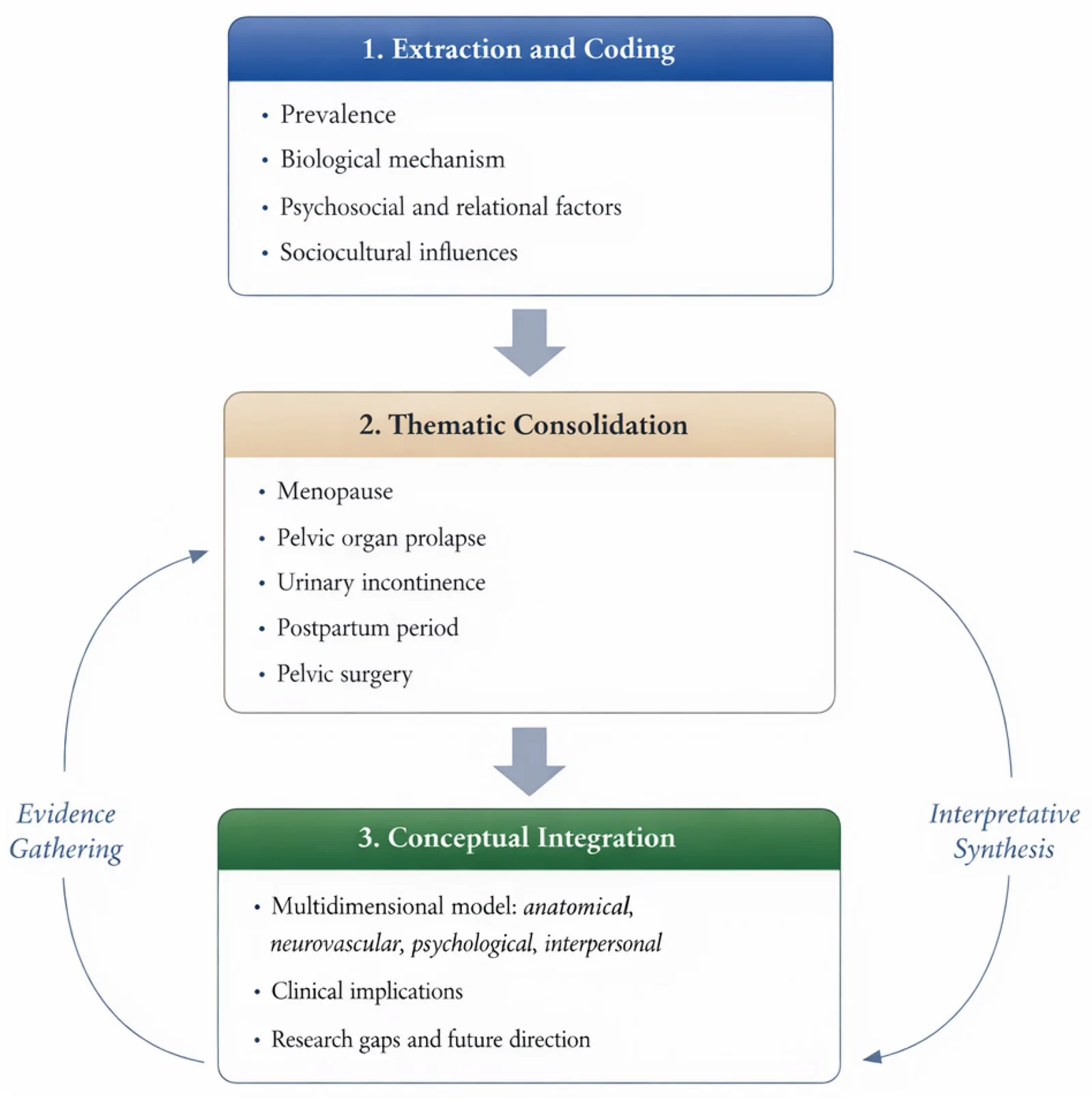
Data Reduction Process.

This step was pivotal in ensuring that the diverse findings related to FSD were transformed into an integrated conceptual framework (Figure 1).

All relevant information from the included studies was meticulously extracted and coded according to the key determinants of FSD: prevalence, biological mechanisms, psychosocial and relational factors, and sociocultural influences. The coded data were then reorganized into five major thematic domains, corresponding to the clinical areas addressed in the review: (1) Menopause, (2) Pelvic organ prolapse, (3) Urinary incontinence, (4) Postpartum period and (5) Pelvic surgery. The five thematic domains were selected according to the topics addressed during a dedicated roundtable discussion on female sexual dysfunction at the International Urogynaecological Association/European Urogynaecological Association 2025 Joint Meeting in Barcelona, which served as the conceptual framework for this review. The final step involved integrating the five thematic domains into a unified interpretative model consistent with the central conclusion of the article.

### 2.4. Selection Process

Two independent reviewers (A.B. and M.S.) independently screened the titles and abstracts of all retrieved records to determine their eligibility for inclusion in the review.

**Figure 2:**
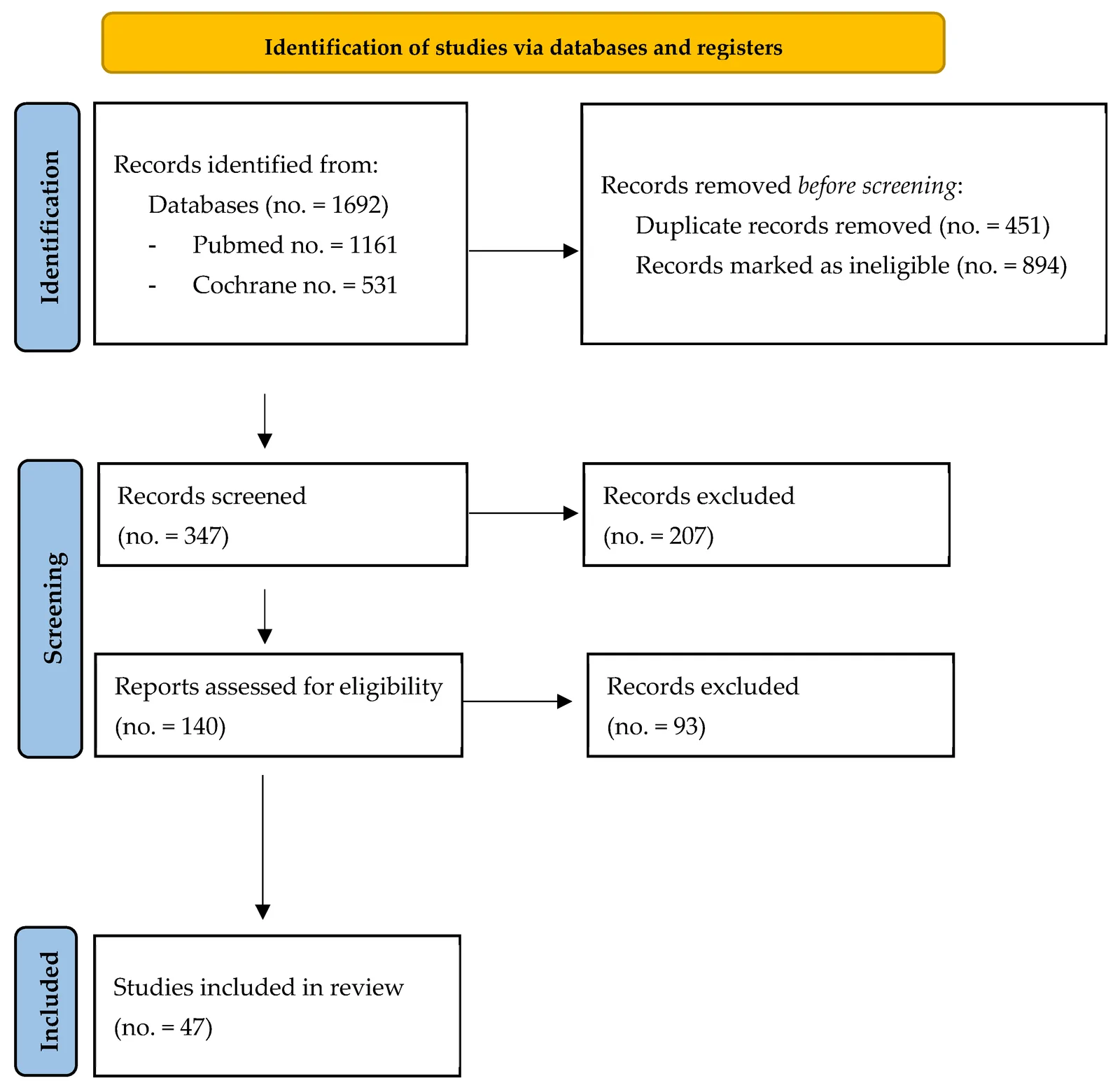
Flow diagram of evidence acquisition.

Disagreements were resolved through discussion and consensus, with consultation from a third reviewer (A.R.) when required. Full-text versions of potentially eligible articles were then assessed against the predefined inclusion and exclusion criteria. Studies that met all eligibility requirements were included in the qualitative synthesis (Figure 2). Subsequently, a narrative synthesis of the evidence was conducted.

## 3. Narrative Analysis

### 3.1. Sexuality in Menopause

Sexuality remains an important component of QoL throughout the lifespan. Contrary to the widespread misconception that sexual interest declines completely with age, a substantial proportion of middle-aged and older women continue to express sexual desire and engage in sexual activity [5]. Data from the Global Study of Sexual Attitudes and Behaviors, involving 27,500 participants from 29 countries aged 40–80 years, showed that 65% of women reported sexual activity (SA) during the previous year, with approximately one in five women over 70 years remaining sexually active [6]. Furthermore, 76% of women considered a satisfying sexual life important for maintaining both a relationship and self-esteem, while only 23% agreed with the statement that older people no longer desire sex. Most participants also supported the use of medical interventions to preserve sexual well-being in later life [6]. Sexual satisfaction remains relatively high among older women. Previous investigations demonstrated that approximately four out of five women between 60 and 80 years reported pleasurable sexual experiences, whereas fewer than 20% reported a lack of enjoyment during intercourse or difficulties achieving orgasm [5]. Although sexual interest often persists during ageing, the menopausal transition is associated with significant changes in SF. Large longitudinal studies have shown that advancing menopausal status negatively influences SA and SF [7,8]. Compared with premenopausal women, peri- and postmenopausal women are more likely to experience reduced sexual desire, impaired arousal and lubrication, and increased dyspareunia, while late perimenopause is associated with a greater risk of vaginal and pelvic pain and a decline in sexual interest [9]. Among these symptoms, vaginal dryness and impaired lubrication appear to be the aspects most consistently associated with advancing age [6]. However, FSD during menopause cannot be explained exclusively by hormonal changes. Increasing evidence suggests that overall health status, frailty, and comorbidities may have a greater impact on SF than chronological age itself [10,11]. Frail women present approximately twice the risk of experiencing deterioration in SF compared with non-frail women. At the same time, both SA and affective relationships have been shown to positively influence QoL and may contribute to reduced frailty, highlighting the importance of sexuality as a component of healthy ageing [10,11]. These findings challenge the common misconception that sexuality becomes irrelevant in later life and emphasize the need for a broader biopsychosocial perspective when addressing sexual health in menopausal women. The influence of psychosocial and relational factors further highlights the complexity of female sexuality after menopause. Sexual behaviours among older women remain diverse and are not limited to vaginal intercourse. A large cross-sectional survey conducted by Herbenick et al. [5] demonstrated that, although sexual intercourse remained the most common partnered SA, other behaviors such as oral sex and mutual masturbation were frequently reported among women over 50 years of age. The use of sex toys and erotic lingerie was also common, while a smaller proportion of women reported activities such as reading erotic material, viewing sexually explicit content, social media flirting, anal sexual practices, or group sex [5]. These data further support the concept that menopausal and older women maintain varied sexual interests and behaviors, underscoring the importance of avoiding age-related stereotypes in sexual health care.

Given the persistence of sexual interest and the high prevalence of sexual concerns during and after menopause, routine assessment of sexual health should be incorporated into clinical practice. Management should adopt a biopsychosocial approach addressing biological, psychological, relational, and social determinants of sexual function. In this context, the Ex-PLISSIT model represents a practical and effective framework for clinical practice [12]. The model encourages healthcare professionals to facilitate discussions about sexuality, provide accurate information regarding age- and menopause-related changes, offer individualized therapeutic suggestions including lubricants and local hormonal treatments, and refer women for specialized sexual therapy when required. Such an approach may help normalize conversations about sexuality in later life and promote individualized, patient-centred care.

### 3.2. The Impact of Pelvic Organ Prolapse on Female Sexual Dysfunction

Pelvic organ prolapse (POP) significantly affects female sexual health through a complex interaction of anatomical, functional, psychological, and social factors. Among women with POP, sexual inactivity is more common than in women with urinary incontinence (UI), with approximately one-third of those with advanced prolapse reporting a negative impact on their sexual relationships [13]. However, the relationship between POP and sexuality is not straightforward. While prolapse may reduce SA and impair aspects of sexual function, overall sexual satisfaction does not always correlate with the presence or severity of prolapse or with its treatment, suggesting that additional psychosocial factors contribute substantially to sexual well-being [13].

Psychological and relational factors play a central role in the sexual experience of women with POP. Recent evidence has identified genital self-image as an important determinant of sexual dysfunction. Women who report negative perceptions of their genital appearance are more likely to experience decreased sexual desire, impaired arousal, and difficulties achieving orgasm [14]. Similarly, concerns regarding a partner’s perception of the prolapse or vaginal appearance may further contribute to sexual distress and avoidance of intimacy. Fear of urinary leakage during intercourse has also been recognized as a major barrier to SA, often resulting in reduced sexual motivation and increased anxiety surrounding sexual encounters [15].

Management options for POP include lifestyle modifications, pelvic floor muscle training (PFMT), vaginal pessaries, and surgical intervention. Conservative treatments may positively influence sexual outcomes. Randomized controlled trials have demonstrated that PFMT can improve sexual function, particularly among women who achieve meaningful gains in pelvic floor muscle strength and endurance [16]. Similarly, sexually active women who successfully use pessaries generally do not experience a decline in sexual function and, in some cases, report improvements [17]. Nevertheless, concerns regarding comfort, partner awareness of the device, and the possibility of displacement during intercourse should be addressed during counselling.

Surgical treatment has been more extensively investigated. Current evidence suggests that transvaginal synthetic mesh and native tissue repairs provide comparable overall sexual function outcomes. However, vaginal mesh procedures appear to be associated with higher rates of dyspareunia when compared with abdominal mesh repairs [18]. These findings highlight the importance of discussing both anatomical and sexual outcomes when counselling women considering surgery.

The assessment of sexual health in women with POP is increasingly challenging because concepts of SA and intimacy continue to evolve. SA is now recognized as encompassing a broad range of behaviours, including foreplay, masturbation, oral sex, and other forms of non-penetrative intimacy, rather than being limited to vaginal intercourse [19]. Consequently, clinicians should acknowledge that sexual activity is self-defined and may vary substantially among individuals.

Many of the questionnaires traditionally used in POP research were validated only in women reporting recent vaginal intercourse, typically within the previous four weeks. As sexual practices become more diverse and the frequency of partnered SA decreases in older populations commonly affected by POP, the validity and inclusiveness of these assessment tools may be limited [20]. Therefore, further validation studies are needed to develop instruments capable of accurately assessing SF and SA across a broader spectrum of sexual behaviours and relationship statuses.

Effective patient-clinician communication remains fundamental for identifying women’s goals, expectations, and concerns regarding sexual health. A patient-centred approach that incorporates regular reassessment and individualized management strategies is essential for optimizing sexual well-being in women living with POP.

### 3.3. The Impact of Urinary Incontinence on Female Sexual Dysfunction

UI is strongly associated with FSD, with up to 45% of affected women reporting sexual difficulties. In general, UI increases the likelihood of impaired sexual function, while pelvic floor muscle (PFM) dysfunction, particularly PFM hypertonia, further increases the risk of sexual disorders [19]. A specific manifestation of UI affecting sexual health is coital incontinence, whose prevalence ranges between 2% and 56%. Leakage occurring during orgasm is commonly associated with detrusor overactivity, whereas urine loss during penetration is typically related to stress urinary incontinence (SUI). Since both conditions may significantly affect SF and QoL, validated questionnaires assessing SF may be valuable during both diagnostic evaluation and follow-up after treatment. Among available therapeutic options, surgical treatment represents the gold standard for women with SUI and is supported by the most robust evidence regarding sexual outcomes. A systematic review including 23 studies identified mid-urethral slings (MUS) as the most frequently investigated procedure. Most studies demonstrated significant improvements in both overall sexual function and individual Female Sexual Function Index (FSFI) domains, together with improved Pelvic Organ Prolapse/Urinary Incontinence Sexual Questionnaire 12 (PISQ-12) scores, particularly following retropubic sling procedures [21]. Single-incision slings (SIS) have also been evaluated in a dedicated systematic review including 13 studies. While coital incontinence often improved or resolved after surgery, new-onset or worsening dyspareunia was reported in a proportion of patients. One of the largest available studies reported dyspareunia rates of 11.7% following SIS compared with 4.8% following MUS, highlighting the importance of balancing potential benefits and adverse sexual outcomes when counselling patients [22]. The evidence supporting pharmacological interventions is less robust. Duloxetine, a serotonin-norepinephrine reuptake inhibitor commonly used for SUI, has rarely been evaluated with SF as a primary outcome. Only one study assessed its effect on sexually active women with SUI, reporting improvements in FSFI scores, QoL, and depressive symptoms after two months of treatment [23]. However, the limited number of studies precludes definitive conclusions. Similarly, pharmacological treatment for overactive bladder (OAB) appears to provide at least partial improvement in self-reported sexual function. Nevertheless, a systematic review of 37 studies concluded that the overall quality of available evidence remains low, limiting the strength of current recommendations [24]. Several emerging treatment modalities have shown promising results, although evidence remains limited or heterogeneous. Energy-based therapies have been increasingly investigated in women with SUI and mixed urinary incontinence with stress-predominant symptoms. However, despite improvements in urinary symptoms, a systematic review including 11 randomized controlled trials found no significant advantages regarding SF when pooled analyses were performed [25]. Bulking agents also represent a promising option. In particular, Serati et al. demonstrated that Macroplastique^®^ injections were safe and effective in improving sexual function and reducing coital incontinence in sexually active women with pure SUI [26]. Nevertheless, additional studies are needed to confirm these findings. Neuromodulation therapies have similarly produced encouraging results. A systematic review evaluating percutaneous tibial nerve stimulation (PTNS) identified three randomized controlled trials and five before-and-after studies, with most reporting improvements in SF [27]. Likewise, intradetrusor botulinum toxin injections have been associated with significant improvements in FSFI scores, although benefits were less evident for pain-related domains [28]. Sacral neuromodulation has also demonstrated positive effects on sexual function in pooled analyses of available studies, although evidence remains relatively limited [29]. Overall, most treatments for UI appear to have a favorable impact on SF and QoL. However, the strength of evidence varies considerably across therapeutic options. Surgical interventions are currently supported by the most robust data, whereas pharmacological treatments, bulking agents, energy-based therapies, and neuromodulation techniques are supported by lower-quality or more limited evidence. Future studies specifically designed to evaluate sexual outcomes are needed to better define the effects of these therapies on female sexual health.

### 3.4. Postpartum Female Sexual Dysfunction

Postpartum FSD is a common and multifactorial condition, affecting between 41% and 83% of women during the first months after childbirth and up to 64% at six months postpartum [30,31]. While childbirth is recognized as the most important risk factor for pelvic floor trauma (PFT), postpartum sexual health is influenced by an interplay of anatomical, hormonal, psychological, and relational factors. Therefore, a comprehensive understanding of postpartum FSD requires a broader perspective beyond pelvic floor injury alone.

Childbirth plays a central role in the development of pelvic floor disorders (PFD), which may subsequently affect SF. Evidence from longitudinal studies demonstrates a strong association between mode of delivery and the risk of PFD. Compared with spontaneous vaginal delivery (SVD), cesarean section (CS) is associated with a lower risk of SUI, OAB and POP, whereas operative vaginal delivery (OVD) is associated with higher rates of anal incontinence and POP [32].

However, the relationship between mode of delivery and SF is less clear. A systematic review and meta-analysis found no significant differences in short- or long-term sexual satisfaction between women undergoing CS and those experiencing SVD [33]. In contrast, accumulating evidence suggests that OVD may negatively affect postpartum SF, although definitive conclusions cannot yet be drawn [34].

Severe perineal trauma represents one of the strongest predictors of postpartum sexual dysfunction. Third- and fourth-degree perineal tears are associated with delayed resumption of SA, higher rates of dyspareunia, and poorer sexual outcomes. A recent systematic review and meta-analysis reported that obstetric anal sphincter injury increased the risk of postpartum FSD approximately threefold and the risk of dyspareunia approximately twofold [35,36]. While episiotomy has been associated with dyspareunia in some studies, current evidence does not support a direct association between episiotomy and overall sexual dysfunction.

Psychological factors also contribute substantially to postpartum sexual health. Women’s subjective experience of childbirth may influence sexual recovery independently of physical trauma. Negative birth experiences, perceived loss of control during labour, and childbirth-related psychological distress have all been associated with postpartum dyspareunia and sexual difficulties [35]. These findings highlight the importance of respectful maternity care, improved communication during labour, and early identification of childbirth-related psychological distress or acute stress disorders. Optimizing the childbirth experience may therefore have long-term benefits for postpartum sexual well-being.

Breastfeeding is another important contributor to postpartum sexual changes. Although breastfeeding provides substantial health benefits for both mother and infant, the associated hormonal milieu may adversely affect sexual function. Elevated prolactin levels suppress ovarian estrogen and androgen production, often resulting in reduced libido, vaginal dryness, and impaired lubrication. The MAMMI study, a longitudinal cohort including 832 primiparous women, demonstrated that six months after childbirth, 46.3% reported reduced sexual interest, 43% reported decreased vaginal lubrication, and 37.5% experienced dyspareunia [37]. Breastfeeding was identified as a significant predictor of postpartum sexual dysfunction, while pre-existing dyspareunia increased the risk of persistent symptoms and vaginal dryness up to 12 months after delivery. Breastfeeding was also associated with dissatisfaction regarding body image [37].

Dyspareunia is among the most frequent postpartum sexual complaints and may arise from multiple interacting mechanisms, including perineal trauma, pelvic floor injury, hormonal changes related to breastfeeding, and psychological factors. The persistence of dyspareunia can significantly delay the resumption of sexual activity and negatively affect overall sexual satisfaction. Given its multifactorial etiology, postpartum dyspareunia should be assessed within a broader biopsychosocial framework rather than being attributed exclusively to obstetric trauma.

Partner-related factors and couple dynamics are often overlooked despite playing an important role in postpartum sexual adjustment. Anxiety regarding intimacy, fear of pain, body image concerns, and changes in family roles may affect both partners and contribute to sexual difficulties. Despite the high prevalence of postpartum sexual problems, these issues frequently remain underrecognized in clinical practice. Only 18% of women report receiving information regarding postpartum sexual changes, and merely 15% discuss sexual concerns with their maternity care providers [38]. For this reason, prenatal counseling involving both women and their partners may help reduce anxiety, improve expectations regarding sexual recovery, and facilitate early recognition and management of postpartum sexual dysfunction [37,39].

Overall, postpartum FSD should be viewed as a multifaceted condition arising from the interaction of pelvic floor trauma, childbirth-related factors, hormonal changes, psychological well-being, dyspareunia, and relationship dynamics. A multidisciplinary and patient-centered approach is therefore essential to optimize postpartum sexual health.

### 3.5. The Impact of Surgery on Female Sexual Dysfunction

POP surgery may have a substantial impact on female sexual health. Current evidence suggests that women with symptomatic prolapse frequently report sexual concerns before surgery, regardless of whether they are sexually active. Common concerns include avoidance of SA because of prolapse symptoms, reduced sexual interest, and concerns regarding intimacy and partner relationships [40,41].

Sexual well-being is a multidimensional construct that extends beyond anatomical correction. Several studies have demonstrated that body image represents an important determinant of SF in women undergoing prolapse surgery. Women with POP often report a negative perception of their genital appearance and reduced self-esteem. Evidence from randomized controlled trials has shown that body image significantly improves following prolapse repair, irrespective of the surgical technique employed, and that improvements in body image are positively associated with improvements in SF [42,43]. The overall effect of prolapse surgery on SF is generally favorable. A large Finnish cohort study including 3515 women undergoing either native tissue repair or mesh-augmented surgery demonstrated significant improvements in PISQ-12 scores at six months after surgery, with benefits maintained for up to five years [44]. Improvements have been reported in several domains of SF, including sexual desire, arousal, and satisfaction, whereas the effects on orgasm and vaginal lubrication remain less consistent across studies. Dyspareunia deserves particular consideration when counseling patients. Existing evidence suggests that pre-existing dyspareunia resolves after surgery in approximately 50% to 74% of affected women [45]. Conversely, de novo dyspareunia appears relatively uncommon, occurring in 0% to 9% of cases overall, although higher rates have been reported following posterior vaginal repairs, where approximately 14% of women may develop new-onset dyspareunia [45].

The impact of uterine preservation on SF remains an area of active investigation. Overall, available evidence suggests that sexual function outcomes are largely comparable between hysterectomy and hysteropexy, whether these procedures are performed for benign gynecological conditions or as part of pelvic organ prolapse repair. Although some variability exists in specific domains such as dyspareunia, arousal, orgasm, and patient satisfaction, RCTs have consistently failed to demonstrate clinically significant differences in overall SF between the two approaches.

In women undergoing hysterectomy for benign indications, a meta-analysis including more than 4000 patients demonstrated that hysterectomy is generally not associated with significant deterioration in overall sexual function, regardless of surgical route [46]. Most studies reported stable or modestly improved postoperative sexual outcomes, particularly regarding pain reduction and sexual desire. However, a substantial subset of women continued to report sexual dysfunction after surgery, and improvements were less pronounced when bilateral salpingo-oophorectomy was performed concomitantly, particularly with regard to lubrication and orgasmic function. Furthermore, no consistent differences have been demonstrated between total and subtotal hysterectomy, while minimally invasive approaches may offer modest advantages compared with abdominal procedures [47,48]. The evidence comparing hysteropexy and hysterectomy in women undergoing prolapse repair is similarly reassuring. The SAVE-U trial [49], which compared sacrospinous hysteropexy with vaginal hysterectomy and uterosacral ligament suspension, demonstrated no significant differences in SF outcomes at two years. Likewise, the SUPeR trial [50] showed comparable improvements in SF and dyspareunia between mesh sacrospinous hysteropexy and vaginal hysterectomy, with these findings remaining stable over long-term follow-up. Other RCTs comparing uterus-preserving procedures, such as the Manchester procedure or sacrospinous hysteropexy, have similarly reported equivalent improvements in SF and low rates of de novo dyspareunia [51].

These findings have been further supported by systematic reviews and meta-analyses, which have not identified significant differences in patient-reported SF between hysterectomy-based prolapse repair and uterus-preserving procedures [52]. Nevertheless, some smaller observational studies suggest that uterine preservation may be associated with greater improvements in selected sexual domains, including desire, arousal, orgasm, partner satisfaction, and body image. These potential benefits may reflect not only anatomical outcomes but also psychological factors related to body integrity, femininity, and personal preferences regarding uterine preservation [53]. Current evidence indicates that both hysterectomy and hysteropexy are associated with favorable sexual outcomes, with no clear superiority of one approach over the other in terms of overall sexual function. Therefore, the choice between uterine preservation and hysterectomy should be individualized, taking into account prolapse characteristics, patient expectations, reproductive wishes, body-image concerns, and informed counseling regarding the benefits and limitations of each surgical option. Overall, prolapse surgery has a predominantly positive impact on SF, body image, and quality of life, with relatively low rates of adverse sexual outcomes when appropriate patient selection and counseling are provided.

## 4. Conclusions

FSD is a complex and multifactorial condition. As highlighted in this review, female sexual health may be affected by different life stages and clinical conditions, including menopause, pelvic organ prolapse, urinary incontinence, childbirth-related pelvic floor trauma, and their respective treatments. Although several sections of this review focus on specific clinical conditions, FSD may also occur in otherwise healthy women and is influenced by a complex interaction of biological, psychological, relational, and sociocultural factors. Current evidence suggests that PFD and menopausal changes can negatively affect SF through physical symptoms, body image concerns, sexual distress, and relationship-related factors. Similarly, the postpartum period represents a particularly vulnerable phase in which pelvic floor injury, hormonal changes, breastfeeding, dyspareunia, and psychological factors may contribute to impaired sexual well-being. While both conservative and surgical treatments frequently improve SF and QoL, outcomes remain highly individualized and depend on the interaction between physical recovery, patient expectations, partner relationships, and psychosocial circumstances.

Despite the high prevalence of FSD, sexual health remains underassessed in routine clinical practice because of sociocultural barriers, misconceptions, and limited patient-provider communication. Therefore, a proactive, patient-centered, and biopsychosocial approach is essential, incorporating counseling, individualized management, and appropriate follow-up. Future research should focus on improving the assessment of FSD through validated and inclusive patient-reported outcome measures that capture not only SF but also sexual distress, relationship factors, and QoL, thereby providing a more comprehensive understanding of women’s sexual health.

## Data Availability

The raw data supporting the conclusions of this article will be made available by the authors on request.

## Abbreviations

The following abbreviations are used in this manuscript:

BMI	Body Mass Index
CS	Cesarean Section
DSM	Diagnostic and Statistical Manual of Mental Disorders
FSD	Female Sexual Dysfunction
FSFI	Female Sexual Function Index
ICD	International Classification of Diseases
MESH	Medical Subject Headings
MUS	Mid-Urethral Sling
NSA	Non-Sexually Active
OAB	Overactive Bladder
OVD	Operative Vaginal Delivery
PFD	Pelvic Floor Disorders
PFM	Pelvic Floor Muscle
PFMT	Pelvic Floor Muscle Training
PISQ-12	Pelvic Organ Prolapse/Urinary Incontinence Sexual Questionnaire 12
PTNS	Percutaneous Tibial Nerve Stimulation
POP	Pelvic Organ Prolapse
RCT	Randomized Controlled Trial
SA	Sexual Activity
SF	Sexual Function
SUI	Stress Urinary Incontinence
SVD	Spontaneous Vaginal Delivery
UI	Urinary Incontinence
WHO	World Health Organization
